# Persisting workarounds in Electronic Health Record System use: types, risks and benefits

**DOI:** 10.1186/s12911-021-01548-0

**Published:** 2021-06-08

**Authors:** Albert Boonstra, Tess L. Jonker, Marjolein A. G. van Offenbeek, Janita F. J. Vos

**Affiliations:** 1 Faculty of Economics and Business, University of Groningen, Groningen, The Netherlands; 2 Customer Service ERP, AFAS Software, Leusden, The Netherlands

**Keywords:** Electronic Health Record, Healthcare organization, Workarounds, Workflow

## Abstract

**Background:**

Electronic Health Records (EHRs) are now widely used to create a single, shared, and reliable source of patient data throughout healthcare organizations. However, health professionals continue to experience mismatches between their working practices and what the EHR allows or directs them to do. Health professionals adopt working practices other than those imposed by the EHR to overcome such mismatches, known as workarounds. Our study aims to inductively develop a typology of enduring EHR workarounds and explore their consequences by answering the question: What types of EHR workarounds persist, and what are the user-perceived consequences?

**Methods:**

This single case study was conducted within the Internal Medicine department of a Dutch hospital that had implemented an organization-wide, commercial EHR system over two years ago. Data were collected through observations of six EHR users (see Additional file 1, observation scheme) and 17 semi-structured interviews with physicians, nurses, administrators, and EHR support staff members. Documents were analysed to contextualize these data (see Additional file 2, interview protocol).

**Results:**

Through a qualitative analysis, 11 workarounds were identified, predominantly performed by physicians. These workarounds are categorized into three types either performed while working with the system (in-system workflow sequence workarounds and in-system data entry workarounds) or bypassing the system (out-system workarounds). While these workarounds seem to offer short-term benefits for the performer, they often create threats for the user, the patient, the overall healthcare organization, and the system.

**Conclusion:**

This study increases our understanding of the enduring phenomenon of working around Electronic Health Records by presenting a typology of those workarounds that persist after adoption and by reflecting on the user-perceived risks and benefits. The typology helps EHR users and their managers to identify enduring types of workarounds and differentiate between the harmful and less harmful ones. This distinction can inform their decisions to discourage or obviate the need for certain workarounds, while legitimating others.

**Supplementary Information:**

The online version contains supplementary material available at 10.1186/s12911-021-01548-0.

## Background

Electronic Health Records (EHRs) have been widely implemented because of their promise of improved patient service, quality, healthcare safety, and reduced costs [1]. EHRs are designed to integrate medical specialties’ specific working routines into one, organization-wide, software application [2]. Unfortunately, reaping these potential benefits continues to be challenging [3, 4] and EHRs’ organization-wide optimal adaptation and use are rare. These systems are often characterized as having a high complexity, highlighting the strong interdependences among health workers. This complexity limits user acceptance [5].

When health professionals experience an EHR system as constraining their activities or blocking their routines, they often devise so-called workarounds: ways to bypass constraints by adapting the EHR system or their use of it [6]. We adopt the following definition of EHR workarounds: *‘workarounds are behaviors that may differ from organizationally prescribed or intended procedures. They circumvent or temporarily ‘fix’ an evident or perceived workflow hindrance in order to meet a goal or to achieve it more readily’* [7, p.2]. In an EHR context, a workaround can involve skipping prescribed steps, entering data that should be entered by others, or registering activities later in the EHR system rather than letting the system guide these activities [8, 9]. Working around an EHR system is common [3, 10] and could have severe consequences, especially given the high interdependence among healthcare workers. Although a recent review showed convergence between explanations for workaround behaviour, it signalled a lack of understanding of the consequences [11, p.352]. Examining the relations between types of workarounds and their consequences is an area in urgent need of additional research [12], for the reasons outlined below.

Whereas researchers tend to distinguish between beneficial and detrimental workarounds [10, 13], others have demonstrated how a single workaround can have both positive and negative implications [14] depending on the perspective taken and the moment of assessment. Understanding the mechanisms between workarounds and their potentially multifaceted consequences is of critical importance for managerial decision making on how to respond to employees’ workarounds [15]. The interwoven patient, professional and financial interests make managerial responses to workarounds also a sensitive issue. Our study aims to offer a typology of persisting EHR workarounds geared to exploring the consequences per distinguished type.

Therefore, we asked *what types of EHR workarounds persist and what are the user-perceived consequences?* We conducted this study at a hospital that had implemented a comprehensive organization-wide EHR system two years before our data collection started and focuses on enduring workarounds that still existed two years after implementation. Through interviews and observations within the hospital’s Internal Medicine department and the EHR system’s support department, workarounds were identified, and the user-perceived consequences were unravelled. Based on these data, we developed a grounded, systematic typology of EHR workarounds and user-perceived consequences.

The study’s contributions are twofold. First, this research advances our understanding of workarounds that persist, which could contribute to creating better fitting EHR systems [16, 17]. Second, by uncovering the specific consequences of workaround types, it provides a basis for managers to decide whether to discourage, temporarily encourage or permanently allow workarounds [12], and prioritize their interventions.

## Theoretical background: types and consequences

Workarounds indicate that working routines are being adopted that differ from the usage prescribed by the system as designed. For example, users may alter their way of working with a system [18] or bypass activities it imposes [19] when they feel that the system constrains rather than supports their workflow. As such, workarounds are user-initiated adjusted working routines that enable the system to fit their personal preferences [20]. By engaging in workarounds, EHR users, explicitly or implicitly, ignore the rules and principles imposed through the system [3] and try to compensate for the system’s shortcomings they perceive.

Following the studies of Patterson [12] and Flanagan et al. [21], attributes of EHR workarounds include: (1) a conflict of goals that need to be addressed in pursuing work practices; (2) a workflow bottleneck that needs to be overcome to carry out a task; (3) the opportunity to conceive a workaround; and (4) the assimilation of humans and technology. The following activities are not considered EHR workarounds: temporary adaptations in response to system downtime, adaptations developed by people other than system users, adaptations developed prior to the implementation of the EHR, and one-time adaptations made in error.

Workarounds in EHR systems may have implications for the quality of care: although they might be harmless, they could also make the difference between life and death [22]. This is illustrated by the following examples of EHR workarounds identified in previous research: not checking the system for medication verification [6, 8, 23, 24], dispensing medication before an order is confirmed in the system [6], and placing copies of patient IDs in several places to bypass the scanning of patient ID wristbands [23].

Workarounds have earlier been classified based on their consequences. For example, Ferneley and Sobreperez [13] categorize workarounds as either *essential*, *hindering* or *harmless*. Another classification logic is seen in the research of Friedman et al. [10] who distinguish temporary versus routinized, unavoidable versus avoidable, and unplanned versus deliberate workarounds.

Taking a negative lens to workarounds highlights that workarounds carry risks for the quality of care, or the professional or financial accountability. First, workarounds may indicate resistance to the routines imposed by the EHR [3]. From this perspective, workarounds can be classified in the same category as making deliberate errors and sabotage, and could jeopardize a system’s efficiency benefits [25]. On the one hand, given that workarounds constitute a divergence between the EHR use as intended by the implementers and the actual use by the healthcare providers, workarounds are a potential source of inefficiencies [20]. They can undermine standardization by not conforming to the system-enforced way of safeguarding patient safety [8]. When staff members bypass a system’s built-in safeguards, workarounds potentially endanger the safety of patients [22], which can potentially lead to increased mortality [39]. Examples are ignoring or disabling specific functions in the EHR system, e.g., security checks [10] or entering data that do not accurately reflect the evolving reality, e.g., by administering medication to the wrong patient or in the incorrect dose [3, 26]. Where EHRs are designed to safeguard the reliability of health information flowing through a healthcare organization, working around these safeguards could lead to unreliable or incomplete patient data [27]. Workarounds may also result in unauthorized access, which can undermine the privacy of patients. In 2019, the Dutch Data Protection Authority (AP) imposed a fine of € 460,000 on the Haga Hospital in The Hague, after more than 80 employees of the hospital had worked around the authorized access to the medical file of a well-known Dutch person who was hospitalized.

From a positive perspective, workarounds indicate system misfits and may initiate improvements to the workflow. Workarounds potentially show areas where the EHR system’s capabilities are not aligned with the healthcare professionals’ needs. Consequently, workarounds can be used to locate misfits between the EHR and the organization, and can thus be considered as a potential source of system improvements [26]. As an illustration, writing allergy-related patient data in a separate note field rather than selecting one of the data options provided by the EHR system might indicate that a fundamental data option is missing [3]. When healthcare providers devise such a workaround, system developers become aware of missing elements, can build in the necessary data entry accordingly, and thereby ensure a better fit between the EHR and the healthcare organization [6]. Workarounds can also be conducted as a quick fix of an unusual problem [28] or conceived creatively [29] to contribute to a well-functioning and normalized workflow [23, 40]. Healthcare professionals can decide to create workarounds in emergency situations if the EHR system does not recognize such urgency in order to be able to continue tasks they consider crucial [27]. In this way, workarounds can be essential for saving lives and property or coping with unexpected circumstances [13], and can save time, reduce medication failures and increase compliance [30].

Despite the clear distinction made by some researchers between positive and negative workarounds [10, 13], workarounds can be double-edged swords. A workaround can have both positive and negative consequences, depending on who, what or when is affected. For example, Koppel et al. [23] encountered users bypassing an EHR’s protocol of double-checking high-risk medication in terms of doses, type and patient. The users saw this workaround as having a positive effect for themselves in terms of a reduction of time and effort allocated to the supply of medications, but a possible negative effect for the patient if receiving the wrong medication or dose. Similarly, Blijleven et al. [3] observed physicians entering patient information in a separate note for colleagues as a response to missing functionality in the EHR. This workaround has positive efficiency implications for those engaging in the workaround, but at the same time has negative consequences for their colleagues who need more time to collect necessary information or for patient’s safety if the separate note is seen [3].

Consequently, a descriptive typology of workarounds (i.e. not in terms of their consequences) is warranted that allows an open-minded yet systematic analysis of the possibly multifaceted consequences of persisting types of work arounds.

## Methods

This research was conducted within the Internal Medicine department and related EHR support team of a Dutch hospital that had implemented an organization-wide commercial standard EHR system. Data were collected through 17 semi-structured interviews with physicians, nurses, administrators, and members of the EHR support staff enriched by observations of six EHR users (see Table 1). Documents were analysed to contextualize these data. All methods were carried out in accordance with relevant guidelines and regulations.

**Table 1:** Details participant interviews and observations

	Code, Professional	Expertise	Duration (min)
Interviews	I_SS2, Support staff	Training facilitator	45
	I_SS3, Support staff	Optimization team	60
	I_SS4, Support staff	Optimization team	60
	I_SS5, Support staff	Order complications	30
	I_SS6, Support staff	Patient registration complications	20
	I_HD1, Help desk	Manager IT Help desk	40
	I_PH1, Physician	Acute medicine	35
	I_PH2, Physician	Nephrologist	40
	I_PH3, Physician	Head of Department, Researcher	60
	I_PH4, Physician	Nephrologist	45
	I_PH5, Physician	Acute Medicine	30
	I_NU1, Nurse	Flex worker	20
	I_NU2, Nurse	Nurse	25
	I_NU3, Nurse	Head Nurse	20
	I_MA1, Medical Admin	EHR core team	45
	I_MA2, Medical Admin	Kidney transplants	25
	I_MA3, Medical Admin	Kidney transplants	25
Observations	O_PH1, Physician	Acute medicine	60
	O_PH2, Physician	Nephrologist	60
	O_PH3, Physician	Head of department	70
	O_PH4, Physician	Nephrologist	70
	O_SS1, Support staff	Solution centre	70
	O_SS2, Support staff	Training facilitator	70

Of these 17 interviews, four were follow-ups with employees observed earlier. In these interviews, the first part was based on the observations to verify the observations and to seek clarifications and reasons for the observed enduring workarounds. All the interviews were semi-structured to leave room for probing. This approach provided the opportunity to not only cover the central themes of this study, but also for new aspects to emerge.

**Fig. 1:**
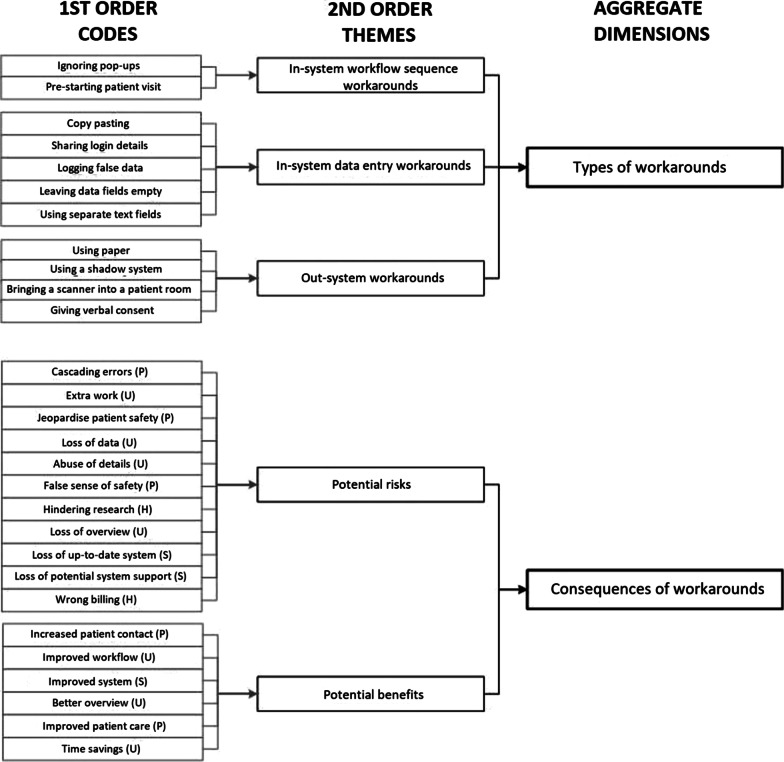
Data structure

Prior to the coding process, relevant documents were read thoroughly, and significant quotes were highlighted. The interview transcripts and observation summaries were then imported into the coding software ATLAS.ti. The coding process itself involved four steps [31]. First, based on the literature review, a sensitizing set of deductive codes was applied. Using these deductive codes, several workarounds, reasons and consequences were identified in the documents and labelled based on earlier work. Second, an open coding approach was used to recode all the transcripts and summaries. The aim was to explore examples of enduring workarounds, the reasons behind them and, specifically, their consequences, other than those identified in earlier research [32]. This resulted in a set of inductive codes (see final codebook in Additional file 3). Each time an inductive code was created, the previously coded documents were re-coded to check whether this code also applied there. By applying this iterative approach, saturation was ensured [33]. Third, similarities and dissimilarities were identified in the codes created in the preceding steps. Here, through axial coding, the codes were grouped and code categories created accordingly, resulting in second-order themes [31, 32]. Existing workaround typologies did not appear suitable to serve as themes because they implied either positive or negative consequences [13], hindering our exploratory efforts, or lacked clear information about the role played by the EHR system [10, 24]. Fourth, through selective coding, relationships between the second-order themes were established and these were then grouped into aggregate dimensions [31]. Figure 1 summarizes the final data structure.

## Results

Based on the data analysis, eleven workarounds were identified and divided into two main groups. The findings reveal a distinction between workarounds used while working within the system (in-system workarounds: workflow sequence and data entry) and workarounds that are used through means other than the EHR to supplement or bypass the system’s practices (out-system workarounds).

### In-system: workflow sequence workarounds

We identified the following two workarounds related to the workflow sequence imposed by the system: ignoring pop-ups and pre-starting a patient’s visit.

#### Ignoring pop-ups

The first workaround is clicking to ignore pop-ups when using the EHR system. This workaround was not seen during the observations but mentioned in interviews by two nurses and three physicians. As one nurse commented: *“There are a few warnings that keep popping up, such as one for allergy verification. These pop-ups occur so often, I hardly notice them anymore. Imagine being in a conversation with a patient, and you want to look something up, and that thing appears on your screen. This means I have to interrupt my conversation to answer this question, which I don’t want, so I close it. At a certain point, you don’t even read what the pop-up says”* (I_NU1). The pop-ups are part of the EHR’s warning system to ensure tasks are executed. However, in practice, the EHR seems to open pop-ups *“at an unworkable and unnecessary frequency”* (I_PH1), which is argued to slow down and be unsupportive of the work process. Physicians *“simply don’t have the time during a consult to look at these things”* (I_PH3). As a nurse explained: *“Look, the problem is that it occurs all the time. If it would pop up twice a year there would be no built-up frustration, and you’d think: Oh right, I do need to ask this again”* (I_NU1).

A potential benefit of ignoring pop-ups for the user is that it preserves a continuous workflow during a consultation: conversations do not have to be interrupted, and time is saved by not responding to the message. However, what follows this workaround, is an expected risk for the patient as a *“false sense of safety”* (I_PH1) is created. The safety protocol built into the EHR is undermined as the nurses and physicians fail to read the warning messages. Further, since ignoring pop-ups becomes a habit, there is a growing risk that the questions posed by the pop-up are *“never asked”* of the patient (I_NU1), which could jeopardize patient safety.

#### Pre-starting a patient’s visit

The second in-system workflow sequence workaround is starting a ‘patient visit’ in the EHR before the patient physically arrives for the appointment. This workaround was observed with two physicians and acknowledged by two others during interviews. Despite the relatively limited supporting data, one physician believed *“this happens a lot”* (I_PH4). In preparation for a patient visit, many physicians want to pre-order treatments such as blood tests to save time during the visit. To achieve this, physicians need to click the ‘start patient visit’ button because this opens a new interface through which orders can be placed. Placing orders is only possible after the ‘visit’ has started and *“this functionality was not there before”* (I_PH4). In effect, the EHR is designed to ensure that tests and medications are not ordered by a physician before patients physically attend their consultation.

The benefits expected of pre-starting a patient’s visit accrue to the user, with one physician explaining that pre-ordering tests improve the workflow during the consultation. On the negative side, once an order is placed, the billing process starts. This means that even if a patient does not show up for their appointment, this workaround will lead to an invoice for the patient because *“blood tests have been registered”* (I_PH4). This increases the administrative tasks for the hospital to ensure that patients do not pay for tests that never took place.

### In system: data entry workarounds

We identified five workarounds related to entering data into the EHR: copy-pasting, using separate text fields, leaving data fields empty, sharing login details, and registering incorrect data.

#### Copy-pasting

The first in-system data entry workaround identified is copy-pasting data from one part of the system to the next, rather than registering data discretely in the system. An example is to select *“a piece of text and copy-paste it into a letter”* (I_PH4). This workaround was mentioned during interviews by three physicians, three members of the support team and by one medical administrator. One reason for this workaround is the perception that *“it will take too much time to register data discretely”* (I_PH4). Another reason is that physicians are *“scared because they are used to working in multiple systems and get nervous about registering in one system and fear losing data”* (I_SS5).

The expected benefit of this workaround is for the user. First, an improved workflow and perceived time savings are achieved when copy-pasting instead of discretely registering the data. Second, a fuller overview is created. As one medical administrator explained: *“Sometimes you don’t know all the medicines being required by patients, so I just take a look at their previous medications to make sure I have the right one* […] *then I can copy-paste that to the in-basket and have the physician prescribe it.* […] *Otherwise, I have to figure it all out myself, Google it and so on.”* (I_MA3).

The likely risks of copy-pasting affect the user, the system, and the hospital in terms of extra work due to the loss of system support as the performers of this workaround miss out on the *“convenience and support* [the EHR] *offers when registering discretely”* (I_PH5), and the hindrance it causes when conducting research with the data present in the EHR. As one physician explains: *“you can create all kinds of reports if the data are registered discretely; you cannot do that if it is only plain text”* (I_PH4).

#### Using separate text fields

The second in-system data entry workaround is using separate text fields in addition to the required data fields in the EHR. This workaround was not seen during observations but mentioned during interviews with three members of the support team, three physicians, and two nurses. A member of the EHR support team commented that she observed this workaround put into practice by physicians during multidisciplinary meetings: *“They are using something called a specialty comment to just to write when they should be discussing the patient.”* (I_SS2).

In some cases, the underlying reason for adding separate note fields is the lack of functionality in the EHR, and it is *“the only option that is available now”* (I_SS2). One nurse explained that if she wants to report a patient’s status, she *“can only enter a fixed number of words or characters”* (I_NU2). The lack of space to make comments creates her need *“to add a separate note”* (I_NU2). Another reason for this workaround is the lack of an overview of the relevant data for a particular physician or specialism: *“*[…] *a problem list is a problem list, but I also see about fifteen other problems which I might not find interesting at all”* (I_PH5). In this situation, the separate note field is used to restructure issues in the problem list. A final reason is the lack of knowledge about the system’s functionality and which is the right button to click. As an alternative, *“they just put it in a note”* (I_SS2).

A possible benefit of this workaround is that, through these separate notes, healthcare providers create a better overview for themselves in the form of a *“good and concise summary of all the relevant information”* (I_PH5). The risks associated with this workaround affect the hospital and the user. Registering data as a separate piece of plain text makes it harder to generate data from the EHR for research purposes. A second risk is that the use of separate note fields leads to a potentially incomplete overview if applied extensively: *“It is a like a toilet roll from which all the sheets have been torn, you have to read through 100 notes? 200 notes? The slightest inconvenience experienced by a patient will have a note allocated to it”* (I_PH3).

#### Leaving data fields empty

We found the third in-system data entry workaround was not entering certain patient-specific information—deliberately leaving data fields empty. This workaround was observed with one physician and mentioned during interviews with five other physicians. A reason for this workaround is that users feel that the EHR restricts their autonomy and directs them to enter data they do not wish to enter: *“I use* [the EHR] *in the way I want to use it, within certain boundaries. I take a certain degree of freedom.* […] *There is always more than one way to skin a cat, and I know we have to work with* [the EHR]*. But, inside* [the EHR]*, I believe everyone should be able to take their own paths, within the frameworks we set along the way”* (I_PH1).

Another related reason is that entering data is time-consuming. A physician mentioned: *“Yesterday I registered a patient that was already known to another specialism. This took me two hours. Two hours to order all this data in the EHR. Imagine that. Normally I would do that too, but I just wrote it down, and it cost me 10 min*” (I_PH3). As is clear from the quote above, not filling in certain data fields in the EHR is expected to benefit the user by saving the physicians time to spend on other activities. On the other hand, there are associated risks for the system and the patient. One physician highlighted that the EHR *“only works well if we all use it correctly”* (I_PH5), meaning that this workaround damages the information quality that the EHR can provide. Moreover, not registering data potentially leads to cascading errors in medicine prescriptions: *“Certain disciplines order an antibiotic treatment and do not include an end date, so it just stays in the system. The patient probably stopped taking the antibiotics a while ago, but the system says otherwise. This, in turn, has consequences for my prescriptions*” (I_PH1).

#### Sharing login details

The fourth in-system data entry workaround identified is sharing login details with other employees, which is a clear violation of hospital policies. This workaround was not observed, and most interviewees denied sharing login details with colleagues. However, five members of the support team recognized this took place: *“I find this very regretful, but I know these things happen”* (I_SS4). Further, a physician explained a situation in which this would occur: *“This is often over the phone: the physician is busy elsewhere. I can imagine that when he picks up the phone and is asked ‘May this patient receive that medicine’, he might say: Just fix it, I’m busy”* (I_PH5).

Most interviewees explained that sharing login details would be done either because of a lack of time or a lack of physical facilities—when *“there is no computer to hand”* (I_NU2). All the interviewees were doubtful that this workaround has overall benefits. One physician felt *“that it saves you a certain amount of work, but that does not stack up against the risks”* (I_PH5). While positive effects were hardly mentioned, the interviewees did identify the risks of sharing login details with co-workers. One member of the support team explained the implications for patient safety: *“Assume that a person with insufficient knowledge orders or gives the wrong medication. Imagine a patient being allergic to a type of medication but receives it anyway. In the worst case, they die. This can happen if you start acting in someone else’s name. It is very dangerous”* (I_SS4).

A risk for the user is that once login details are shared, there is no control over potential abuse of these details, *“you never know if these details will be used again without your knowledge”* (I_PH1).

#### Entering incorrect data

The final in-system data entry workaround is entering data that does not represent reality. This workaround was not observed, but described by a physician and a nurse. As an illustration, the physician explained: *“I received the exact same pop-up as four weeks ago, to pay extra attention to the patient’s medication. Now I can’t continue, I have to enter new data into the system. Otherwise I cannot prescribe medicines. So what happens is, you are just going to make it up, you know, you just want to get on”* (I_PH1). The nurse in mentioning this workaround recognized it in the following situation: *“even though I emptied the catheter bag at 11 pm, I enter this as emptied at 9.59 pm”* (I_NU1).

In the first instance, this workaround was used deliberately to bypass the restricting power of the system over the work process to avoid delays in ordering medicines. As a physician explained: *“*[The EHR] *should not rule over us, it should help us. I mean, we need to use* [the EHR]*, but it should not be the case that it dictates how I should do my job”* (I_PH1). In the second, the nurse explained she has no other option than to use this workaround as in the EHR *“a day needs to be finalized at 10 pm* […] *That’s just how it is designed”* (I_NU1).

In the nurse’s case, there is an expected benefit of registering incorrect data for the users: it avoids a distorted image of the previous day improving workflow the next day. All activities scheduled for the previous day need to be registered by 10 pm to avoid *“other alarm bells starting to ring”* (I_NU1). The nurse did not expect this workaround to bring about dangerous situations since *“30 min or an hour’s difference is negligible”* (I_NU1). The physician said that, as with ignoring pop-ups, entering incorrect data may lead to a false sense of safety, negatively affecting the patient.

### Out-system workarounds

In addition to the several workarounds that are used within the EHR system described above, our data also demonstrate that people bypass the EHR system by using other systems or relying on other routines. These out-system workarounds include writing down information on paper, using one or more shadow systems, giving verbal consent for dispensing medication, and detaching a scanner from the COW (EHR- Computer-on-Wheels) to take it into a patient’s room.

#### Using paper

First, many interviewees recognized the use of paper for making notes. This workaround was observed once and mentioned twelve times during interviews with nurses, physicians, members of the support team and medical administrators. In effect, users rely on paper in combination with the EHR: *“When I do my round of patient visits, I always have a piece of paper with me.* […] *I would rather write some keywords on paper, sit behind my desk, think about it, and register the information in peace.”* (I_PH1). We also observed that nurses write patient information on their hands with the intention to enter it into the EHR at a later stage.

As described above, the physician feels that first writing down information on paper and later registering it in the EHR helps to process the information, suggesting a lack of trust in their own abilities to directly register the data correctly. Another motive for using paper is to maintain eye contact with patients. As one nurse explained: *“The patient might be very nervous, in such cases, you want to make eye contact. An option would be to bring your computer, but then you would be talking to the screen instead of to the patient.* […] *You look less at the patient’s face, so you don’t see the impact of what you are saying.* […] *And the patient could feel less heard”* (I_NU1).

The expected benefits of paper are for the user and for the patient. First, the user saves time that can be used to process and reconsider the information provided by the patient. Second, patient contact is preserved. Risks for the user are a loss of overview, extra work and a potential loss of data as paper might *“get lost or lie around”* (I_NU3). Further, patient safety is jeopardized as there is a possibility *“to overlook items because they are not on the work lists on your computer, especially if you don’t know that patient well”* (I_NU2).

#### Using shadow systems

The second out-system workaround identified is the use of a system other than the EHR. Two physicians, four members of the support team, and one medical administrator admitted to using Microsoft Word and Microsoft Excel either as a substitute for or as a complement to the EHR.

A reason given for using these shadow systems was the lack of overview presented by the EHR: *“Every day there are three, four, five notes added for each patient. These are all separate, so I cannot just scroll through them. So, open, close, open, close. Then I also have to remember each note’s content. Therefore, I open a Word document next to it, to create my own file”* (I_PH3). Another reason for using shadow systems is a lack of functionality in the EHR: *“*[The EHR] *does not support planning intakes.* [The planners] *keep an Excel file with the entire planning, while you really just want to be able to do this in the EHR”* (I_SS5). A final reason for this workaround is that the healthcare provider prefers a different layout than that proposed by the EHR: *“People make the entire letter layout in Word because they find letters generated by the EHR ugly”* (I_SS5).

An expected benefit for the users when using shadow systems is that better overviews will be created. Also, by keeping an Excel file, the intake planners can schedule patient intakes a few months ahead, which they could not do if they only use the EHR system, thereby improving their workflow. In terms of the system, by acknowledging the deficiencies of the EHR, *“improvements in the system can be made”* (I_SS5). On the downside, shadow systems have the expected risk for users of creating extra work when physicians have to enter information in both the EHR and their shadow system. Also, there is a risk of forgetting to register data in the EHR alongside the shadow system, resulting in the EHR system not being up-to-date.

#### Giving verbal consent for dispensing medication

The third out-system workaround identified is a physician giving verbal consent to a nurse for dispensing medication, only to also order this medication in the EHR sometime later. This workaround was not observed but mentioned during interviews with two nurses and one physician: *“What I sometimes try, and it depends a bit on the nurse to be honest, is that I say: here, you have my consent to carry it out. I will register the order later, or e-mail me at the moment that I have to order it. That's how I do it”* (I_PH2). The physician continued by outlining situations in which this would happen, which are often due to inconveniences: *“Imagine standing in the corridor, and you receive such a call, well, then you don’t have* [the EHR] *at hand. Or you’re in the middle of an outpatient visit and you are phoned. Right, it is always unexpected”* (I_PH2). That is, giving verbal consent occurs because of a lack of time or physical facilities. One of the nurses offered a third reason for this workaround: *“no time, no motivation.* […] *If they* [physicians] *are at home and don’t feel like starting up the system they tend to give consent verbally”* (I_NU3). As such, she is implying that this workaround is related to a physician’s willingness to order through the EHR directly.

Expected benefits of this workaround for the user are an improved workflow and time savings. For the patients, better care is expected to follow a verbal consent: *“I think patient care is paramount, before the administrative part”* (I_NU3). There is a possible risk of this workaround for the system. If one forgets to record the verbal request for medication in the EHR later, then *“according to the system, this patient did not receive the medication”* (I_NU3), leaving the system not up-to-date.

#### Separating a scanner from its Computer-on-Wheels (COW)

The final out-system workaround entails detaching a scanner from its ‘computer-on-wheels’ (COW) to scan the wristbands of patients and the labels on infusion bags. The COW is a fairly large input/output device that forms part of the EHR.

Nurses are supposed to bring the COW into the patient room and scan each infusion bag for every patient separately while monitoring the COW’s screen. This workaround was not observed but admitted by a nurse during an interview, who performs this workaround to not *“wake up patients by the COW’s noise”* (I_NU3).

The expected benefit of this workaround is that patients are not disturbed by the COW during the night. However, taking the scanner into the patient room and away from the COW, might jeopardize patient safety as the nurse will be unable to notice any errors that might appear on the screen when accidentally *“scanning the wrong infusion bag, or the wrong patient”* (I_NU3).

Overall, we were able to identify 11 workarounds. Two of them were in-system workflow sequence workarounds, five in-system data entry workarounds and four out-system workarounds. Each of the outlined workarounds were used by one or more occupational groups within the hospital. The expected consequences for each of these workarounds entailed benefits as well as risks and affected the user, the patient, the hospital, or the system. Table 2 summarizes these findings.

**Table 2:** Workarounds and user-perceived benefits and risks

Category	Workaround	Identification	Benefits	Risks
In-system workflow sequence workarounds	Ignoring pop-ups	Interview (5x) NU, PH	Improved workflow (U), time savings (U)	Jeopardize patient safety (P), false sense of safety (P)
	Pre-starting a patient’s visit	Observation (2x), interview (2x) PH	Improved workflow (U)	Incorrect billing (H)
In-system data entry workarounds	Copy-pasting	Observation (2x), interview (7x) PH, SS, MA	Improved workflow (U), time savings (U), better overview (U)	Extra work (U), loss of potential system support (S), hindering research (H)
	Using separate text fields	Interview (8x) NU, PH, SS	Better overview (U)	Hindering research (U), loss of overview (U)
	Leaving data fields empty	Observation (1x), interview (5x) PH	Improved workflow (U), time savings (U)	Loss of potential system support (S), cascading errors (P)
	Sharing login details	Interview (5x) SS	Improved workflow (U)	Abuse of details (U), jeopardize patient safety (P)
	Entering incorrect data	Interview (2x) NU, PH	Improved workflow (U)	False sense of safety (P)
Out-system workaround	Using paper	Observation (1x), interview (12x) NU, PH, SS, MA	Improved workflow (U), increased patient contact (P)	Extra work (U), loss of data (U), loss of overview (U), jeopardize patient safety (P)
	Using shadow systems	Interview (6x) PH, SS, MA	Better overview (U), improved workflow (P), improvements of the system (S)	Extra work (U), system not up-to-date (S)
	Giving verbal consent for dispensing medication	Interview (3x) NU, PH	Improved workflow (U), time savings (U), improved patient care (P)	System not up-to-date (S), cascading errors (P)
	Separating a scanner from its COW	Interview (1x) NU	Improved patient care (P)	Jeopardize patient safety (P)

Codes in identification column indicate who reported the workaround: NU = nurse; PH = physician; SS = support team member; MA = medical administrator. Codes attached to benefits and risks indicate to whom the users think these consequences apply: U = user; P = patient; S = system; H = hospital

## Discussion

**Fig. 2:**
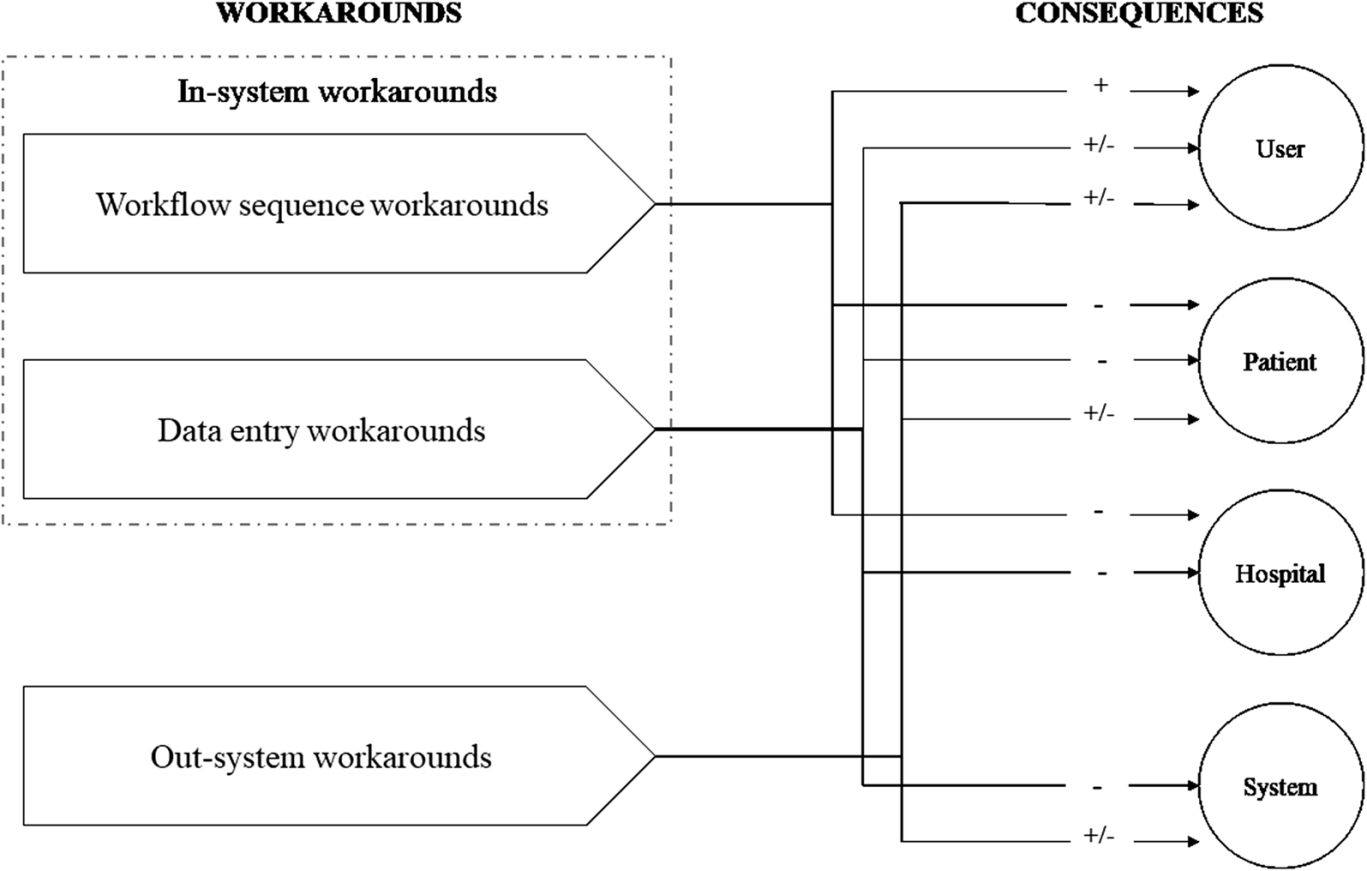
Types of workarounds and perceived consequences

This study’s aim was to develop a typology of EHR workarounds and explore their user-perceived consequences by answering the question: what types of EHR workarounds persist and what are the user-perceived consequences? Thus, we focused on enduring rather than on temporary workarounds. These workarounds persisted for two main reasons. First, many workarounds in this hospital signal that individual physicians and medical departments have the professional autonomy to deviate from system-enforced and prescribed work processes. Doctors are ultimately accountable for patients and can, for example, prescribe medicines by telephone and have them included in the EHR system afterwards if this is in the direct medical interest of the patient. Second, EHR systems can structurally hinder desired and established work processes, requiring adaptations that the EHR supplier does not support. In the latter case, health professionals and their departments can resolve the problem by explicitly accepting and institutionalizing a workaround. Sometimes it is also necessary to first treat a patient and update the system for completeness, reimbursement, or research. From this perspective, while a workaround may be highly legitimate, especially in acute and emergency situations, it requires post-hoc data registration and processing [41]. Below we discuss how the identified workarounds types relate to user-perceived risks and benefits. Figure 2 summarizes the outcomes.

### Three types of EHR workarounds and consequences

We have identified three types of workarounds, two in-system and one out-system workarounds. The *in-system workflow sequence workarounds* are created in response to the time consuming and impositional characteristics of the EHR. Expected consequences of this type of workaround are benefits for the user (improved workflow; time savings), risks for the patient (jeopardized safety; false sense of safety) and for the hospital (incorrect billing).

Second, *in-system data entry workarounds* are created in response to a lack of time, fear of losing data, lack of knowledge, lack of functionality; lack of overview and the impositional features. The expected benefits for the user following this type of workaround are an improved workflow, time savings and a better overview. However, these workarounds also have foreseen risks: (1) for the user, such as additional work, loss of overview, excessive details, loss of potential system support; (2) for the patient, such as jeopardizing safety, false sense of safety and cascading errors; and (3) for the hospital, such as hindering research.

The third type, *out-system workarounds,* are responses to a lack of trust in one’s own abilities, reduced patient contact, lack of time, and limited motivation. Out-system workarounds have expected benefits for users (improved workflow; better overview), for patients (increased patient contact; improved patient care), and for the system (improvement of the system). However, out-system workarounds also carry possible risks for the users, such extra work, loss of overview and loss of data, for the patient, such as jeopardizing safety and cascading errors, and for the system, such as the system not being up-to-date.

### Consequences of workarounds

First, the data show that users expect each type to have consequences for both users and patients. In terms of the potential benefits, users recognize six different positive consequences. An improved workflow was most often mentioned. This benefit is expected to follow from 9 of the 11 workarounds, mainly from the in-system workflow sequence and in-system data entry workarounds. As such, most of the reported workarounds are seen to contribute to an easier and more flawless workflow for users. Turning to risks, the study’s participants mentioned 11 different risks, which were more evenly distributed across the workaround types. Jeopardizing patient safety was seen as the most common risk involved. Surprisingly, the interviewed users did not acknowledge any consequences of out-system work arounds for the hospital, while they showed themselves aware of the adverse effects for the system’s integrity. Likewise, they did not think in-system workflow workarounds would affect the system, but did see risks for the hospital. Consequences for themselves and for patients may be more concrete or meaningful for them than those affecting the hospital or the system. For persisting workarounds it will be important to also systematically weigh the hospital- and system-related benefits and risks. Therefore, more research on user attributions regarding their workarounds is called for.

Second, this study shows how users do not perceive their workarounds to produce only benefits or only risks. Indeed, this study reveals how seven workarounds are expected to yield both benefits and risks for the same stakeholder, mainly the users. Other workarounds are perceived to have both beneficial and risky implications but for different stakeholders. Importantly, overall the workaround types that persist remain controversial: while users expect to benefit from all workaround types, each type is also expected to create risks, especially for patients on the longer term and for research purposes.

### Instigators of workarounds

Only 2 of the 11 identified workarounds were not created by physicians. These were *Bringing only a scanner into the patient room* and *Sharing login details,* which were instigated by nurses and support staff. Since scanning medication, blood bags, and infusion bags is part of the nurses’ tasks [7], we can assume it is unlikely that this workaround will also be employed by physicians. None of the interviewed physicians admitted sharing login details with other employees, although the interviewed support team members did mention that they had observed physicians engaging in this workaround. A likely explanation for the physicians’ reticence to admit this workaround is that sharing login details potentially violates the confidentiality of patient health information [34]. Since this *“unethical and dangerous”* act [34, p.177] would have to be deliberate, physicians might be reluctant to acknowledge it openly.

Our results suggest that nurses engage in fewer types of workarounds than physicians. This is surprising, because earlier research has claimed that nurses are *“masters of workarounds”* [7, p.2]. A plausible explanation, given that age has been shown to play a key role in intentions regarding EHR use [35], is that the participating nurses in our study were considerably younger than the participating physicians. As such, our contrary findings may be due to the age pattern of our respondents rather than a genuine divergence from earlier findings. Finally, nurses may engage in few workarounds, but employ these frequently as the examples above constitute repetitive tasks.

### Implications

This study contributes to the literature on EHR workarounds by offering a typology of enduring workarounds and their perceived consequences, specifically. Despite the fact that EHR systems are designed to meet a hospital’s needs, initial misfits between the system and the organization occur [36], leading to workarounds in the adoption phase. We show three main types of workarounds that persist after the adoption phase and asked users to reflect on their expected consequences. Packaged software systems were introduced as a solution to avoid the high costs of customized software implementations [37]. However, the persisting workarounds and user-perceived risks involved, raise the question whether the post-adoption costs outweigh the avoided customization costs, when the risks of persisting workarounds cannot be mitigated. This seems an area for future research.

This study enriches the literature by taking a health professionals’ perspective on EHR workarounds, whereas other studies have applied a managerial [38] or a system perspective [6]. These earlier studies, by looking from a perspective other than that of the health professional, fail to unravel users’ perceptions and awareness of the consequences of their actions.

In practical terms, the results show the need to make health professionals more aware of the possible wider consequences of certain workarounds. While the health professionals in this study perceived that all three types of workarounds could negatively affect some patients, they seemed to have a partial awareness of organizational and system consequences. To mitigate the risks illustrated in this paper, hospitals could invest in resolving the reasons behind the most harmful workarounds. Theoretically, resolutions can be directed at the user, the EHR system or the organization of work [42]. If physicians can easily prescribe and order through mobile apps integrated with the EHR system, regardless of their location, this would obviate the need for some out-of-system workarounds. Some users may stick to their initially invented workarounds, while system improvements have been realized. Here, continuing education and support may help. Organizational resolutions can relate to (1) adapting authorizations, (2) changing the task distribution between physicians, nurses, and administrators, (3) reducing the required data registration, or 4) shifting registration tasks to specific employees, such as scribes.

### Limitations and future research

There are some limitations that may have affected this study’s results. First, while a clear set of workarounds have been identified, some may have been overlooked. It is quite possible that not all workarounds were admitted or even recognized by the interviewees. As one physician commented: *“A lot will happen behind the scenes which no one will find out about.* […] *I think a lot of people conduct workarounds and just think it is part of the job”* (I_PH4). This may have limited the quantity and the range of workarounds presented in this paper. However, the proposed three main types seem sufficiently robust for transfer to other contexts. Second, this study was conducted at the Internal Medicine and supporting departments of a large hospital. Healthcare professionals in other departments or hospitals may create other workarounds for different reasons. Further, teaching hospitals, as this one, tend to have more elaborated EHRs [3]. This could have influenced the type of workarounds, and their expected consequences, reported in this paper. Future researchers should be aware of this when applying these results in smaller hospitals.

Considering the types of workarounds, the results record only a few in-system workflow sequence workarounds compared with in-system data entry and out-system workarounds. As such, the workarounds identified in this study are not predominantly responses to perceived misalignments in the workflow. The users primarily created workarounds to deal with data registration rather than because the system was unsupportive of their workflow. This indicates that healthcare professionals deliberately work around EHR systems in order to avoid the extra administrative tasks that come with such a system, or as a form of resistance to information technology in general. This could be a relevant area for future research. Further, given the exploratory method used in this study, future research could focus on different medical specialties or on healthcare organizations other than hospitals. Finally, given the possibility that users did not voice all the workarounds they enact, we would suggest that future research on EHR workarounds employ direct and preferably relatively unobtrusive observations when examining the creation and application of workarounds, e.g. through participatory observation.

## Conclusions

This study has increased our understanding of the persistence of working around Electronic Health Records through a typology of enduring workarounds coupled with their user-perceived risks and benefits. Our typology can promote awareness among EHR users and hospital managers of the different types of workarounds and enable them to distinguish harmful from less harmful workarounds. This may support them in their decisions to prohibit, discourage or obviate the need for certain workarounds, while encouraging and possibly institutionalizing others.

## Supplementary Information

**Additional File 1**. Observation scheme. **Additional File 2.** Interview protocol. **Additional File 3**. Codebook.

## Data Availability

The datasets used and analysed during the current study are available from the corresponding author upon reasonable request.

## Abbreviation

EHR: Electronic Health Record
