# Supplementary material for: Persisting workarounds in Electronic Health Record System use: types, risks and benefits
Source: BMC Med Inform Decis Mak. 2021 Jun 8;21:183. doi: 10.1186/s12911-021-01548-0 (PMC8186102; doi:10.1186/s12911-021-01548-0)
Supplement: Supplementary file 1 — Additional File 1. Observation scheme. Additional File 2. Interview protocol. Additional File 3. Codebook. [file 12911_2021_1548_MOESM1_ESM.docx]

# Title Page

Persisting workarounds in Electronic Health Record System use:
types, risks and benefits

## Authors

Albert Boonstra^1*^, Tess L. Jonker^2^, Marjolein A.G. van Offenbeek^1^ & Janita F.J. Vos^1^

1. Faculty of Economics and Business, University of Groningen, The Netherlands
2. Customer Service ERP, AFAS, Leusden, The Netherlands

*Correspondence to [Albert.Boonstra@rug.nl](mailto:Albert.Boonstra@rug.nl)

# Additional File 1: Observation scheme

Observant displays a different way of working than is prescribed in the hospital’s Governance for Data Registration

Yes

No

No

No

Additional notes

………………………………………………………………………………………………

Observant asks members of the support team for tips/tricks to improve workflow using the EHR

Yes

No

No

No

Additional notes

…………………………………………………………………………………………………

Member of the support team intrudes into the observant’s way of working to express possible improvements to the way of working with the EHR

Yes

No

No

No

Additional notes

…………………………………………………………………………………………………

Member of the support team introduces a workaround as a tip/trick to improve workflow using the EHR

Yes

No

No

No

Additional notes

…………………………………………………………………………………………………

Observant expresses opinion about the EHR

…………………………………………………………………………………………………

# Additional File 2: Interview protocol

General

1. Please explain your main role at the hospital and daily activities as related to the EHR?
2. What is your personal opinion about the EHR?

Workarounds, reasons for workaround, possible consequences

1. Case A: During a patient visit, the healthcare provider takes notes on paper instead of directly entering the data into the EHR.

a. What is your first thought after reading this scenario?

b. What could be possible positive consequences of this method?

Follow up: Who will be affected by these consequences?

c. What could be a possible negative consequence of this method?

Follow up: Who will be affected by this consequence?

1. Case B: A nurse believes that a patient needs medication, but is not allowed to order this in the EHR (they are not authorised in the EHR to perform that action). As a solution, the doctor in question shares their login details with the nurse so that they can order the medication.

a. What is your first thought after reading this scenario?

b. What could be a possible positive consequence of this method?

Follow up: Who will be affected by this consequence?

c. What could be a possible negative consequence of this method?

Follow up: Who will be affected by this consequence?

The previous two cases are examples of so-called “workarounds”. As explained earlier, workarounds are ways of working with the system other than those prescribed or requested by the EHR.

1. Could you think of workarounds that are used in your work environment?

a. What could be a possible positive consequence of this workaround?

Follow up: Who will be affected by this consequence?

b. What could be a possible negative consequence of this workaround?

Follow up: Who will be affected by this consequence?

c. Repeat this question until no more examples can be thought of.

# Additional file 3: Codebook

*Note*: D = deductive code; I = inductive code.

| *Descriptive code* | *Representative quotation* |
| --- | --- |
| In-system: data entry workaround | |
| Copy-paste (D) | “What a whole lot of physicians do is instead of discretely registering, they just select a piece of text and copy-paste it into their letters.” (I_PH4) |
| Share login details (D) | “It takes time. This is often over the phone, the physician is often someplace else busy with something. I don’t know in what department this happens, but I can imagine that when one picks up the phone and is asked ‘May this patient have that medicine’ he might say ‘Just fix it because I’m busy’.” (I_PH5) |
| Entering false data (I) | “We can enter fluid balances in the EHR and you have to do that within one day. The EHR will "click" to the next day at ten o'clock in the evening: this was one day. So then, even if you empty the catheter bag at eleven in the evening, you enter it as 9:59 PM.” (I_NU1) |
| Not filling in data fields (I) | “I am against all that registering. Everything needs to be registered. […] I will start registering when I see the purpose of it. […] Look, if I need it, I will ask it then. What is the purpose of registering language? […] I don’t need that information, I just want to know the exception, well, I know those by heart.” (I_PH2) |
| Separate text field (I) | “A problem list is a problem list and then you also have 15 other problems that I might not find interesting at all. I also understand that as a doctor you need a compact overview of the problems in your field. So, yes, it has to do with the fact that you cannot filter properly for just your own information in the EHR. And that can even change per patient. So that's why you make a sort of summary for yourself, yes that's actually what you do in those notes.” (I_PH5) |
| In-system: workflow sequence workaround | |
| Ignoring pop-ups (I) | “There are a few warnings that keep popping up, for example the one concerning allergy verification. These pop up so often, that you become blind to them. Imagine being in a conversation with a patient, you want to look something up and that thing appears on your screen. This would mean I have to interrupt my conversation to ask this question, which I don’t want, so I close it. At a certain point you don’t even read what the pop-up says anymore” (I_NU1) |
| Pre-start patient's visit (I) | “There is a workaround to saying the patient is not here yet, but I will already start his visit in the EHR so I can enter my orders.” (I_PH4) |
| Out-system workaround | |
| Using pieces of paper to make notes (D) | “I prepare my outpatient clinic. I do that a few days in advance in order not to be surprised and then have to sort things out. I know a lot, but then I just make old-fashioned notes on an A4 sheet, points of interest that are also important at the time of consultation at the emergency shelter and I just fill it in on paper.” (I_PH1) |
| Using a shadow system (D) | “We have an Excel file for the donors, screening lists with when they will arrive. Someone just had to be placed in between others, so I have to paste and copy everything. We also keep track of things like that from donors. We plan the screening and send it to the patients. We do that in an Excel file for the two-day screening.” (I_MA2) |
| Bringing scanner into patient room (I) | “When I have to hang a new infusion bag for a patient, for example, I will not take the COW with me into the corridor. This is because it just makes a lot of noise. So what I do then is that I take the scanner with me. Then I always hope that it goes well, scan the patient and scan the bag and that you get no system errors that you cannot continue and that way.” (I_NU3) |
| Giving verbal consent (I) | “What I sometimes try, and this depends a bit on the nurse, I say that very honestly, then I say: here you have my assignment and carry it out, then we will place the order later, or send me an e-mail in a moment, that says I have to order later. Or I give it because it is something acute, I will create that order within half an hour. That's how I do it.” (I_PH2) |
| Expected risks of workaround | |
| Cascading errors (D) | “People register things they in fact did not do. And then people compound mistakes from their colleagues.” (I_PH3) |
| Extra work (D) | “It entails a lot of double work. There are a couple of notes that you can pull into another note very easily. It is sad to hear that people keep copy-pasting.” (I_SS5) |
| Jeopardize patient safety (D) | “A nurse is not educated to order certain medications so doesn't know whether there will be any complications or whatever. Imagine giving someone two medications that form a poisonous mixture.” (I_SS5) |
| Loss of information (D) | “I you forget to write things down or you leave it for too long, then the information could be a little less complete.” (I_PH1) |
| Abuse of details (I) | “You never know whether these details will be used again without your knowing.” (I_PH1) |
| False sense of safety (I) | “So what happens is that you just fill in something, you know, you just want to continue your work. But yes, it creates a false sense of safety.” (I_PH1) |
| Hindering research (I) | “If you do not register precisely, which is part of the agreement, there are lots of things we cannot do in the future, reports or ensure the quality of research or whatever.”(I_PH2) |
| Loss of overview (I) | “It is a like a toilet roll from which all the sheets have been torn, you have to read through 100 notes? 200 notes? The slightest inconvenience experienced by a patient will have a note allocated to it.” (I_PH3) |
| Loss of up-to date data (I) | “The advantage of such a system is that you can have real-time information. That’s the advantage of a generic work process. It is disappointing this doesn’t happen.” (I_SS6) |
| Loss of potential system support (I) | “The EHR only works properly is everyone uses it well, and that’s not happening in this hospital right now.” (I_PH5) |
| Wrong billing (I) | “If someone doesn’t show up but you make it seem like they did by pre-starting a patient’s visit, then billing starts.” (I_PH2) |
| Expected benefits of workaround | |
| Increased patient contact (D) | “I make time for my patients, I can allocate time to them, explain things, ask things. While, when I have to type things, it takes me so much more time [...] and through writing [on paper] I can use that time to spend talking to my patient.” (I_PH1) |
| Improved workflow (D) | “I mean it enabled them to track the patients that they wanted to track, make sure they were able to follow up on them, and kind of like have a place where it is more manual but at least it is not a piece of paper which you write everybody’s name on.” (I_SS4) |
| Time savings (D) | “A positive consequence would be that the physician is saving time because they do not have to place the order now.” (I_SS2) |
| Improvement of the system (D) | “We have become much more critical towards the EHR.” (I_SS6) |
| Better overview (I) | “I think it helps them look at the different therapies they are giving to these patients, see how these cases are evolving, discuss it as a group and it enables them to follow up on it instead of having these things falling through the cracks.” (I_SS4) |
| Improved patient care (I) | “I think it has to do with the fact that if your patient is in pain, you want them to feel comfortable. Patientcare comes first, before the administrative part.” (I_NU3) |
